# Исследование стероидного профиля слюны методом высокоэффективной жидкостной хроматографии с тандемным масс-спектрометрическим детектированием у детей с врожденной дисфункцией коры надпочечников

**DOI:** 10.14341/probl13551

**Published:** 2025-12-02

**Authors:** М. А. Тюльпаков, Н. Ю. Калинченко, Д. В. Быченков, В. А. Иоутси, М. А. Анцупова, А. Р. Елфимова, Е. В. Нагаева

**Affiliations:** Национальный медицинский исследовательский центр эндокринологии имени академика И.И. ДедоваРоссия; Endocrinology Research CentreRussian Federation

**Keywords:** ВДКН, слюна, ВЭЖХ-МС/МС, альтернативный биоматериал, стероидный профиль, CAH, saliva, HPLC-MS/MS, alternative biomaterial, steroid profile

## Abstract

**ОБОСНОВАНИЕ:**

ОБОСНОВАНИЕ. Лечение врожденной дисфункции коры надпочечников (ВДКН) подразумевает подбор минимально эффективной дозы гидрокортизона, по данным стероидного профиля сыворотки, что требует многократных внутривенных заборов крови. Уровни стероидных гормонов, определяемые иммуноферментным анализом, зачастую не отражают их истинную концентрацию в сыворотке в силу имеющихся ограничений метода. Кроме того, возможной причиной получения неточных данных может быть «стрессорная реакция» на процедуру забора венозной крови, что особенно актуально в детском возрасте. С целью преодоления данной проблемы в настоящее время разрабатываются альтернативные малоинвазивные методы оценки стероидного профиля у пациентов с ВДКН. В серии зарубежных исследований в качестве многообещающего альтернативного биоматериала предложена слюна. Использование слюны, в том числе с возможностью ее сбора в домашних условиях, позволяет не только исключить «стрессорный фактор» во время венепункции, повысив тем самым точность анализа, но и снизить нагрузку на средний медицинский персонал.

**ЦЕЛЬ:**

ЦЕЛЬ. Оценка корреляции между концентрациями стероидных гормонов в крови и слюне в пубертатной и допубертатной группе пациентов с ВДКН методом высокоэффективной жидкостной хроматографии с тандемным масс-спектрометрическим детектированием (ВЭЖХ-МС/МС).

**МАТЕРИАЛЫ И МЕТОДЫ:**

МАТЕРИАЛЫ И МЕТОДЫ. Одномоментное интервенционное исследование методов диагностики и компенсации заболевания у 45 пациентов с установленным диагнозом «ВДКН (классический дефицит 21-гидроксилазы)» в возрасте от 3 до 17 лет. Всем детям проведено одномоментное лабораторное исследование в ГНЦ РФ ФГБУ «НМИЦ эндокринологии» Минздрава России в октябре 2024 г.

**РЕЗУЛЬТАТЫ:**

РЕЗУЛЬТАТЫ. В допубертатной группе (N=14) выявлена сильная положительная корреляция между концентрациями стероидов в соответствующих образцах сыворотки и слюны для концентрации 17ОН-прогестерона (р<0,001; r=0,88), андростендиона (р<0,001; r=0,84) и дигидроэпиандростерона (р=0,001; р=0,78). В пубертатной группе (N=31) выявлена сильная положительная корреляция между концентрациями стероидов в соответствующих образцах сыворотки и слюны для концентрации 17ОН-прогестерона (р<0,001; r=0,94), андростендиона (р<0,001; r =0,94), тестостерона (р<0,01; r=0,94) и прогестерона (р<0,001; r=0,79), а также умеренная корреляция для 21-дезоксикортизола (р=0,003; r=0,52).

**ЗАКЛЮЧЕНИЕ:**

ЗАКЛЮЧЕНИЕ. Для большинства стероидов, используемых для диагностики и контроля проводимой терапии у пациентов с ВДКН, выявлена сильная или умеренная корреляция между концентрациями стероидов в соответствующих образцах сыворотки и слюны.

## Abstract (en)

**BACKGROUND:**

BACKGROUND: Currently, alternative minimally invasive methods for assessing the steroid profile in patients with congenital adrenal hyperplasia (CAH) are being actively developed. Saliva has proven to be a promising medium in a series of international studies. The use of saliva as an alternative minimally invasive biological material not only reduces the stress associated with venipuncture but also decreases the workload for medical staff, as saliva collection can be performed at home.

**AIM:**

AIM: Evaluate the correlation between concentrations of steroid hormones in the blood and saliva in prepubertal and pubertal groups of patients with congenital adrenal hyperplasia using high-performance liquid chromatography with tandem mass spectrometric detection (HPLC-MS/MS).

**MATERIALS AND METHODS:**

MATERIALS AND METHODS: A prospective intervention study for diagnosing and disease compensation in 45 patients aged 3 to 17 years undergoing a routine inpatient examination with an established diagnosis of CAH (classic 21-hydroxylase deficiency). All children underwent a one-stage laboratory test at the National Medical Research Center of Endocrinology of the Ministry of Health of the Russian Federation in October 2024.

**RESULTS:**

RESULTS: In the prepubertal group (N=14), a strong positive correlation was found between serum and saliva for the concentration of 17OH-progesterone (p=<0.01; r=0.88), androstenedione (p=<0.001; r=0.84) and dehydroepiandrosterone (p=0.001; r=0.78). In the pubertal group (N=31), a strong positive correlation was found between serum and saliva for the concentrations of 17OH-progesterone (p=<0.01; r=0.94), androstenedione (p=<0.01; r=0.94), testosterone (p=<0.01; r=0.94), and progesterone (p=<0.01; r=0.79), as well as a moderate correlation for 21-deoxycortisol (p=0.003; r=0.52).

**CONCLUSION:**

CONCLUSION: Most steroids used for diagnosis and monitoring the therapy in patients with CAH have a strong or moderate correlation between serum and saliva.

## ОБОСНОВАНИЕ

В настоящее время общепринятыми маркёрами оценки адекватности терапии врожденной дисфункции коры надпочечников (ВДКН) у детей являются уровни 17-гидроксипрогестерона, андростендиона и тестостерона в сыворотке крови методом иммуноферментного анализа (ИФА) [1][2]. Данный метод имеет ряд ограничений: неудовлетворительную специфичность, включающую возможность завышения результатов из-за «перекрестной реакции» с другими близкими по строению стероидами, такими как 17-гидроксипрегненолон, либо их занижение в результате так называемого хук-эффекта [3][4][5]. Кроме того, данная методика не позволяет одновременно оценивать концентрацию других андрогенов, которые являются важными маркерами адекватности подбора терапии и ограничивается определением только одного показателя за один анализ [6].

Высокоэффективная жидкостная хроматография с тандемным масс-спектрометрическим детектированием (ВЭЖХ-МС/МС) может устранить эти недостатки. Преимущества метода ВЭЖХ включают хроматографическое разделение стероидов, что минимизирует или полностью исключают интерференции со сходными по строению соединениями, а масс-спектрометрия обеспечивает высокую селективность и чувствительность за счет идентификации молекул по их молекулярным массам. Использование метода MRM (multiple reaction monitoring) в тандемной масс-спектрометрии повышает селективность и позволяет точно количественно анализировать множество стероидов в ходе одного анализа [7][8]. Это значительно расширяет возможности мониторинга терапии ВДКН в рамках одного исследования. Кроме того, возможной причиной завышения результатов может быть «стрессорная реакция» на забор венозной крови, что особенно актуально для пациентов детского возраста. С целью преодоления данной проблемы разрабатываются малоинвазивные методы оценки стероидного профиля [9–12]. Один из таких методов предполагает использование слюны в качестве биологического материала для определения стероидов методом ВЭЖХ-МС/МС [13–16].

Существует несколько методик сбора слюны, например: пассивное слюнотечение и применение тампонов для сбора образца [17]. В ходе ранее проведенного на базе нашего центра сравнительного анализа методов сбора слюны у пациентов с ВДКН установлено, что использование сорбирующих материалов для сбора слюны в большинстве случаев приводит к занижению результатов [18]. Также было установлено, что наиболее предпочтительным методом сбора слюны для оценки стероидного профиля является «пассивное стекание» в полипропиленовые пробирки. Более того, в группе пациентов с ВДКН и здоровых добровольцев проведена оценка корреляции между концентрациями стероидов в соответствующих образцах крови и слюны. Выявленная в результате исследования корреляция для некоторых, критически важных для диагностики и коррекции терапии при ВДКН андрогенов, оказалась умеренной или слабой, что может быть обусловлено малой выборкой и требует увеличения численности групп пациентов с ВДКН [18].

## ЦЕЛЬ ИССЛЕДОВАНИЯ

Целью исследования стала оценка корреляции между концентрациями стероидных гормонов в крови и слюне в пубертатной и допубертатной группах пациентов с ВДКН методом ВЭЖХ-МС/МС.

## МАТЕРИАЛЫ И МЕТОДЫ

## Место и время проведения исследования

ГНЦ РФ ФГБУ «НМИЦ эндокринологии» Минздрава России (далее: ФГБУ «НМИЦ эндокринологии»).

## Время исследования

Октябрь 2024 г. 

## Изучаемые популяции (одна или несколько)

Популяция: пациенты детского возраста, проходившие плановое стационарное обследование в ФГБУ «НМИЦ эндокринологии» с установленным диагнозом «ВДКН (классический дефицит 21-гидроксилазы)».

Критерии включения: пациенты до 17 лет включительно с диагнозом «Врожденная дисфункция коры надпочечников дефицит 21-гидроксилазы», проходившие плановое стационарное обследование в ФГБУ «НМИЦ эндокринологии».

Критерии исключения: пациенты без врожденной дисфункции коры надпочечников.

## Способ формирования выборки из изучаемой популяции

Сплошной способ формирования выборки.

## Дизайн исследования:

## Методы

Для корреляционного анализа между концентрациями стероидов в образцах слюны и сыворотки методом ВЭЖХ-МС/МС образцы соответствующего биоматериала были собраны у 45 пациентов с установленным диагнозом «ВДКН (классический дефицит 21-гидроксилазы)» в возрасте от 3 до 17 лет (медиана 13) обоих полов, проходящих плановое стационарное обследование и получающих заместительную терапию глюкокортикоидами (гидрокортизон, преднизолон, дексаметазон) и минералокортикоидами (флудрокортизон). Согласно оценке полового развития были сформированы 2 группы: допубертатная и пубертатная. Характеристики обеих групп представлены в таблице 1. Два родных брата допубертатного возраста (младше 9 лет) были распределены в пубертатную группу в связи с наличием пубертатной стадии полового развития, что было подтверждено высоким уровнем ЛГ в ходе стимуляции аналогами ГнРГ. Образцы слюны собирались методом «пассивного стекания» натощак, в полипропиленовые пробирки типа «эппендорф», с предварительным полосканием рта водой за 10 минут до сбора образца. Затем слюна из собранных образцов была аликвотирована в полипропиленовые пробирки объемом от 450 до 950 мкл (в зависимости от собранного объема), которые после этого подвергались заморозке при -80 °С в центре биобанкирования ФГБУ «НМИЦ эндокринологии». Перед анализом образцы размораживали при комнатной температуре и центрифугировали; для анализа использовали супернатант.

**Table:** Таблица 1. Характеристика пациентов в допубертатной (N=14) и пубертатной (N=31) группах

Признак	Допубертатная группа (n=14)	Пубертатная группа (n=31)
N	Me [ Q1; Q3] / n (%)	N	Me [ Q1; Q3] / n (%)
Пол	Мужской	14	4 (29%)	31	16 (52%)
Женский	10 (71%)	15 (48%)
Форма	Сольтеряющая	14	12 (86%)	31	23 (74%)
Вирильная	2 (14%)	8 (26%)
Возраст (недель)	14	62 [ 38; 103]	31	172 [ 78; 215]
Доза ГК (мг/кг/сут) по гидрокортизону	14	6,4 [ 0; 26,5]	31	19,1 [ 0; 26]

Непосредственно после сбора слюны у пациентов производили забор венозной крови в пробирки с активатором сгустка, которые затем центрифугировали для получения сыворотки. Сыворотку аликвотировали в отдельные пробирки и также замораживали при температуре -80 °С. Перед анализом сыворотку размораживали при комнатной температуре и перемешивали на шейкере.

## Оборудование и реагенты

Аналитические стандарты: в качестве внешних стандартов в работе были использованы 11-дезоксикортизол (Steraloids, USA), 17-гидроксипрогестерон (Steraloids, USA), кортизол (Steraloids, USA), кортизон (CIL, USA), 21-дезоксикортизол (Steraloids, USA), андростендион (Steraloids, USA), дегидроэпиандростерон (Steraloids, USA), тестостерон (Steraloids, USA), прогестерон (Steraloids, USA); в качестве внутренних стандартов были использованы кортизол-d4 (CIL, USA), прогестерон-¹³С3 (CIL, USA), дегидроэпиандростерон-d6, 17-OH-прогестерон¹³C3 (CIL, USA), 11-дезоксикортизол-d5 (CIL, USA), 21-дезоксикортизол-d8 (CIL, USA), кортизон-d7 (CIL, USA).

Реагенты: метанол (для УФ, ИК, ВЭЖХ, МС, титрования, EvaScience, Россия), этилацетат (Sigma-Aldrich, for LC-MS, Germany). Деионизированная вода была приготовлена в системе водоочистки Milli-Q Advantage A10 (Millipore, Франция), сульфат цинка (99,0-100,5%, EMPROVE, Merck, Германия).

Материалы для сбора слюны: пробирки типа эппендорф (SHANGYU YITE PLASTIC LIMITED, Китай), центрифужные пробирки (Thermo Scientific, Corning, США).

Материалы для сбора крови: пробирки с разделительным гелем для сыворотки BD Vacutainer® SST™II Advance (Becton Dickinson, Нидерланды).

Оборудование: для анализа использовалась система ВЭЖХ-МС/МС, состоящая из хроматографа Agilent 1290 Infinity II (Agilent Technologies, США), укомплектованного четырехканальным насосом со смесителем низкого давления, автосемплером и термостатом колонок, а также масс-спектрометра AB Sciex QTrap 5500 (AB Sciex, Сингапур) c источником ионизации TurboV, способного работать в режиме химической ионизации при атмосферном давлении (ХИАД). Хроматографическое разделение проводили на колонке xSelect HSS С18 2,5 мкм, 3,0×75 мм с размером частиц 2.5 мкм (Waters, Ирландия).

## Пробоподготовка образцов слюны

Пробоподготовку всех образцов слюны проводили в соответствии с методикой, описанной нами ранее [18]. К 500 мкл слюны добавляли 5 мкл внутреннего стандарта и 900 мкл этилацетата. После перемешивания и центрифугирования отбирали верхний органический слой, упаривали досуха и растворяли в 110 мкл смеси метанола и воды (30:70) перед анализом.

## Пробоподготовка образцов сыворотки крови

Пробоподготовку образцов сыворотки проводили аналогично методике, описанной ранее [19]. В аликвоту сыворотки объемом 300 мкл добавляли 30 мкл внутреннего стандарта, 150 мкл 0,1M раствора сульфата цинка и 200 мкл метанола. После перемешивания и центрифугирования надосадочную жидкость упаривали до удаления органического растворителя, после добавляли 500 мкл этилацетата. После перемешивания и центрифугирования отбирали верхний органический слой, упаривали досуха и растворяли в 110 мкл смеси метанола и воды (30:70) перед анализом.

## Условия хроматографического разделения

Хроматографическое разделение проводили в градиентном режиме с использованием двухкомпонентного градиента на основе метанола и воды. Скорость потока составляла 0,4 мл/мин, температура колонки 40 °C, объем пробы — 80 мкл. Для разделения использовали градиентную программу с изменением содержания обоих компонентов подвижной фазы: повышение содержания метанола от 50 до 60% с начала анализа за 6 минут, затем повышение до 90% за 3 минуты и выдержка при этих условиях в течение 2 минут, после возвращение к условиям начала анализа за 1 минуту и уравновешивание колонки в течение 3 минут.

## Условия масс-спектрометрического детектирования

Для детектирования хроматографического потока использовали трехквадрупольный масс-спектрометр, оснащенный источником химической ионизации при атмосферном давлении. Ионизацию компонентов проводили в режиме регистрации положительных ионов, ток иглы коронного разряда устанавливали на значение 5 мкА, давление газа в области распыления составляло 50 psi (фунт/дюйм²), газа-завесы 28 psi, температура нагревателя источника 500 °C. Регистрация компонентов осуществлялась в режиме мониторинга множественных реакций (multiple reaction monitoring, MRM), работающем в заданных временных интервалах. Временной интервал для каждого компонента составлял 90 сек., время сканирования всех MRM-переходов — 1 сек. Параметры были подобраны для каждого компонента индивидуально. Управление, сбор и обработку количественных данных осуществляли при помощи программного обеспечения Analyst 1.6.3 (AB Sciex).

## Статистический анализ

Статистический анализ выполнен с помощью программного обеспечения SAS OnDemand for Academics. Описательная статистика количественных признаков представлена в виде медиан, первых и третьих квартилей; категориальных — в виде абсолютных и относительных частот. Корреляционный анализ выполнен с помощью метода ранговой корреляции Спирмена. Уровень статистической значимости принят равным 0,05.

## Этическая экспертиза

Исследование одобрено локальным этическим комитетом «НМИЦ эндокринологии №4 от 22.02.2023.

## РЕЗУЛЬТАТЫ

Был проведен корреляционный анализ между концентрациями стероидов в образцах крови и слюны в допубертатной и пубертатной группах (табл. 2).

**Table:** Таблица 2. Корреляция исследуемых стероидов в образцах крови и слюны в допубертатной (N=14) и пубертатной (N=31) группах

Пубертатная группа
Параметр 1	Параметр 2	N	p, метод Спирмена	r
17-OHПрогестерон Слюна	17-OHПрогестерон Сыворотка	31	<0,001	0,94
11-Дезоксикортизол Слюна	11-Дезоксикортизол Сыворотка	31	0,002	0,54
21-Дезоксикортизол Слюна	21-Дезоксикортизол Сыворотка	31	0,003	0,52
Андростендион Слюна	Андростендион Сыворотка	31	<0,001	0,94
Тестостерон Слюна	Тестостерон Сыворотка	31	<0,001	0,87
Кортизол Слюна	Кортизол Сыворотка	31	<0,001	0,72
Кортизон Слюна	Кортизон Сыворотка	31	<0,001	0,74
Прогестерон Слюна	Прогестерон Сыворотка	31	<0,001	0,79
ДГЭА Слюна	ДГЭА Сыворотка	31	0,067	-
Допубертатная группа
17-OHПрогестерон Слюна	17-OHПрогестерон Сыворотка	14	<0,001	0,88
11-Дезоксикортизол Слюна	11-Дезоксикортизол Сыворотка	14	0,235	-
21-Дезоксикортизол Слюна	21-Дезоксикортизол Сыворотка	14	0,069	-
Андростендион Слюна	Андростендион Сыворотка	14	<0,001	0,84
Тестостерон Слюна	Тестостерон Сыворотка	14	0,002	0,67
Кортизол Слюна	Кортизол Сыворотка	14	0,568	-
Кортизон Слюна	Кортизон Сыворотка	14	0,375	-
Прогестерон Слюна	Прогестерон Сыворотка	14	0,345	-
ДГЭА Слюна	ДГЭА Сыворотка	14	0,001	0,78

В результате корреляционного анализа стероидного профиля в допубертатной группе выявлена сильная положительная корреляция между концентрациями стероидов в образцах сыворотки и слюны для: 17ОН-прогестерона (р<0,001; r=0,88), андростендиона (р<0,001; r=0,84) и дигидроэпиандростерона (р=0,001; р=0,78) (рис. 1).

**Figure:**
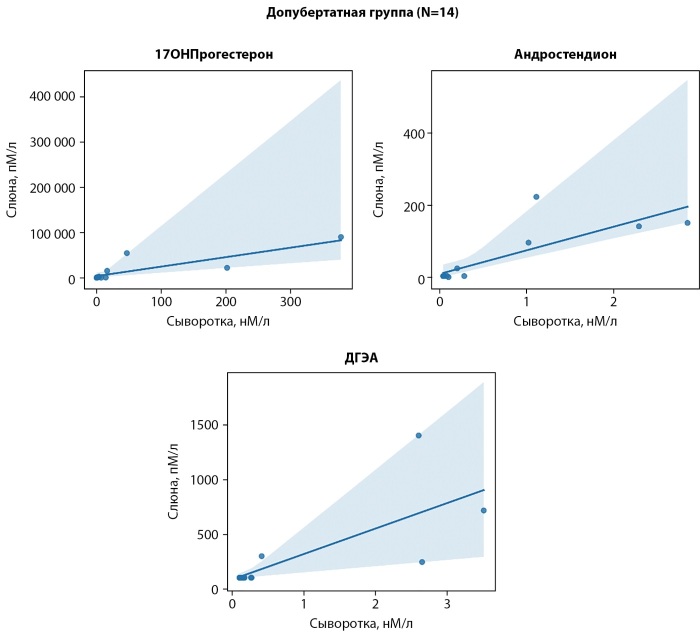
Рисунок 1. Корреляция стероидов между кровью и слюной в допубертатной группе.

В пубертатной группе выявлена сильная положительная корреляция между концентрациями стероидов в образцах сыворотки и слюны для 17ОН-прогестерона (р<0,001; r=0,94), андростендиона (р=<0,01; r=0,94), тестостерона (р<0,001; r=0,94) и прогестерона (р<0,001; r=0,79), а также умеренная корреляция для 21-дезоксикортизола (р=0,003; r=0,52) (рис. 2).

**Figure:**
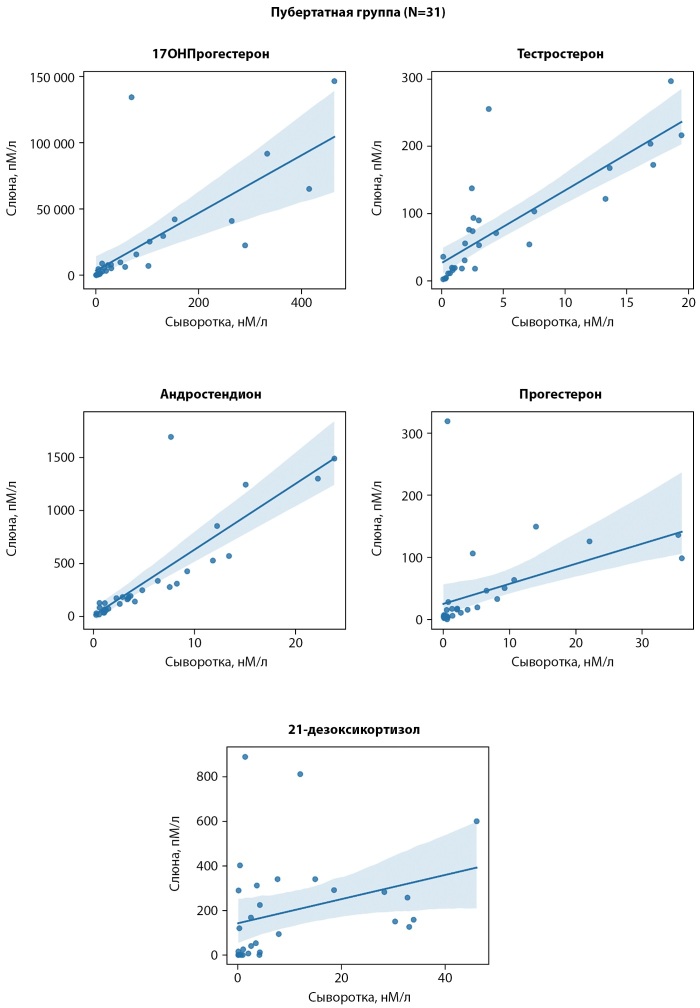
Рисунок 2. Корреляция стероидов между кровью и слюной в пубертатной группе.

## ОБСУЖДЕНИЕ

Использование слюны для определения стероидного профиля у пациентов с ВДКН в качестве альтернативного биоматериала в сравнении с сывороткой крови обладает рядом преимуществ, таких как: исключение «стрессорной реакции» при венепункции и снижение нагрузки на средний медицинский персонал, так как образцы слюны могут быть собраны самостоятельно пациентом в домашних условиях. Как любой, метод определения стероидов в слюне обладает рядом недостатков: трудности сбора слюны в определенных возрастных группах (дети раннего возраста), низкие концентрации интересуемых стероидов в слюне [17][20].

В рамках данного промежуточного исследования возможности использования слюны как альтернативного биоматериала для диагностики и оценки компенсации ВДКН, мы получили сопоставимые с ранее опубликованными исследованиями зарубежных авторов результаты для концентраций 17ОН-прогестерона, тестостерона и андростендиона [13][14].

Исходя из полученных результатов, можно сделать вывод о том, что для большинства интересующих нас стероидов имеется сильная положительная корреляция между концентрациями стероидов в образцах сыворотки и слюны. По всей видимости, отсутствие сильной корреляции для 21-дезоксикортизола в пубертатной группе связано с низкой выборкой пациентов.

В настоящее время имеются референсные интервалы гормонов стероидного профиля сыворотки для различных возрастных групп. В перспективе, исходя из коэффициентов корреляции или из регрессионных моделей, мы планируем вывести формулу перерасчета референсных интервалов концентрации стероидных гормонов для сыворотки в соответствующие показатели для слюны, однако, отсутствие нормального распределения данных при текущем размере выборки, требуется существенного увеличения ее объема.

## ЗАКЛЮЧЕНИЕ

Для большинства стероидов, используемых для диагностики и контроля терапии пациентов с ВДКН, выявлена сильная или умеренная корреляция между концентрациями стероидов в соответствующих образцах сыворотки и слюны. Для выведения формулы перерасчета референсных интервалов концентрации стероидных гормонов сыворотки в соответствующие показатели слюны необходимо расширение выборки пациентов.

## ДОПОЛНИТЕЛЬНАЯ ИНФОРМАЦИЯ

Источники финансирования. Данная работа была финансово поддержана Министерством здравоохранения Российской Федерации, исследование образцов слюны у детей проводилось в рамках государственного задания № 123021000036-2.

Конфликт интересов. Авторы декларируют отсутствие явных и потенциальных конфликтов интересов, связанных с содержанием настоящей статьи.

Участие авторов. Все авторы одобрили финальную версию статьи перед публикацией, выразили согласие нести ответственность за все аспекты работы, подразумевающую надлежащее изучение и решение вопросов, связанных с точностью или добросовестностью любой части работы.

Благодарности. Работа выполнена с использованием материалов Уникальной научной установки «Коллекция биологического материала пациентов с эндокринными патологиями» ГНЦ РФ ФГБУ «НМИЦ эндокринологии им. академика И.И. Дедова» Минздрава России (Москва, Россия).
